# Effect of a Compound Energy Field with Temperature and Ultrasonic Vibration on the Material Properties and Bending Process of TC2 Titanium Alloy

**DOI:** 10.3390/ma14237192

**Published:** 2021-11-25

**Authors:** Tiejun Gao, Kaifeng Wang, Zhiyuan Ling, Zhongjin Wang

**Affiliations:** 1 College of Aerospace Engineering, Shenyang Aerospace University, Shenyang 110136, China; kaifeng_wang@126.com; 2 China Hangfa Dongan Engine Co., Ltd., Harbin 150066, China; zhiyuan_ling@163.com; 3 School of Materials Science and Engineering, Harbin Institute of Technology, Harbin 150001, China; wangzj@hit.edu.cn

**Keywords:** compound energy field with temperature and ultrasonic vibration, TC2 titanium alloy, mechanical properties, bending properties, microstructure

## Abstract

Due to the low formability and forming quality of titanium alloy, the forming process of a compound energy field (CEF) with temperature and ultrasonic vibration was proposed. Tensile tests were carried out to investigate the effect of the CEF on the true stress–strain curve, yield strength, elastic modulus, and other mechanical properties of the TC2 titanium alloy. Bending tests assisted by CEF were also performed to investigate the effect of different parameters of the CEF on bending force, spring-back, bending fillet radius, and microstructure of TC2 titanium. The results demonstrate that compared to the process under a single-temperature field, the CEF can reduce yield strength, elastic modulus, bending force, bending fillet, and the spring-back angle, which shows that the CEF can further increase the high-temperature softening effect of TC2 titanium. Furthermore, this effect becomes more remarkable when ultrasonic vibration energy increases. As a result, the formability of titanium alloy can be improved.

## 1. Introduction

With the continuous improvement of modern aircraft requirements for operational performance and safety performance, airframe structure materials have evolved from the original stage of wood and cloth to the new stage of aluminum alloys, titanium alloys, superalloys, composite materials, and other materials over the past 100 years. In the process, the material requirements and the function requirements mutually prompt each other. Titanium alloys have numerous advantages over other metal materials, including low density, high strength, high- and low-temperature resistance, superior corrosion resistance, and good compatibility with composite materials [1,2,3,4,5]. As a result, titanium alloys play an irreplaceable role compared to other metals.

Since the 1950s, when titanium alloys were initially utilized in aircraft fuselages, the use of titanium alloys in aircraft and engines has increasingly grown. For example, the C-5 transport aircraft, which entered service in 1970, used 5% titanium alloy, and the C-17 transport aircraft, which entered service in 1992, used 10% titanium alloy. In addition, titanium alloy is used at a rate of 26% and 41% in the B2 strategic bomber and the F22 fighter plane, respectively.

According to published data, using titanium alloy instead of aluminum alloy increases the heat resistance temperature of an aircraft’s surface from 200 °C to 400 °C [6,7]. Utilizing titanium alloy instead of stainless steel also reduces an engine’s weight by 40–50% [8]. As a result, titanium alloy has become one of the most popular materials in modern aircraft design.

In addition to having outstanding properties, titanium alloys have high strength, low elastic modulus, and low elongation [9], and are considered to be hard-to-deform materials. It has become a big challenge to form components with complicated forms using traditional methods. As a result, thermoforming is currently the most-used forming process. Due to the softening effect caused by high temperature, forming resistance decreases and formability improves when the sheet is heated to a particular temperature (usually over 500 °C; the superplastic state should surpass 900 °C) [10,11,12,13,14,15,16].

Zhu et al. studied the V-shape bending tests of TC4 titanium. They discovered that when the temperature exceeded 500 °C, spring-back was significantly reduced [17]. Guang et al. investigated the forming limit of TC4 titanium. The best temperature for forming is between 550 and 700 °C [18]. Lang et al. conducted drawing tests and discovered that when the temperature was raised to 600 °C, formability improved considerably [19].

Ultrasonic vibration-assisted forming is a process in which ultrasonic vibration with a certain direction and energy is applied to a specimen or mold to improve formability. As early as 1955, Blaha et al. carried out ultrasonic vibration-assisted tensile tests on single-crystal zinc. It was observed that the flow stress reduced with the superposition of ultrasonic vibrations. This phenomenon is called the “softening effect” (Blaha effect), which was the earliest description of the effect of ultrasonic vibration on the plastic-forming process of metal. Langenecke et al. [20] used single-crystal zinc to conduct a tensile test assisted by ultrasonic vibration in 1966. The results revealed that ultrasonic vibration causes a softening effect, which is similar to thermal softening caused by high temperatures [21]. As investigation of ultrasonic vibration-assisted forming moves forward, it may be discovered that ultrasonic vibration not only makes the material’s forming force [22,23] and the friction coefficient between the mold and the specimen lower [24], but also enhances the material’s forming performance and quality [25,26,27,28,29]. In particular, the power of the ultrasonic generator capacity has improved in recent years; ultrasonic vibration energy can now be employed in a considerably larger range of applications. Ultrasonic vibration-assisted forming has been used in the processes of blanking, deep drawing, bending, and bulging for metals such as steel, aluminum alloys, magnesium alloys, and titanium alloys. For small or micro-sized parts, ultrasonic vibration-assisted forming has unique advantages over other processes [30,31].

According to the above findings, heating or applying ultrasonic vibration to a specimen can increase its formability. As a result, if an ultrasonic vibration energy field is introduced into the titanium alloy-forming process at a specific temperature to combine the temperature field and ultrasonic vibration, the superposition of the thermal-softening effect of high temperature and the “softening effect” caused by ultrasonic vibration may further improve the formability and quality of the titanium alloy. Thus, this paper clarifies the effect of the CEF on the bending properties of titanium by analyzing the true stress–strain curve, yield strength, elastic modulus, bending force, spring-back, and bending fillet radius.

## 2. Bending Process Analysis

**Figure 1:**
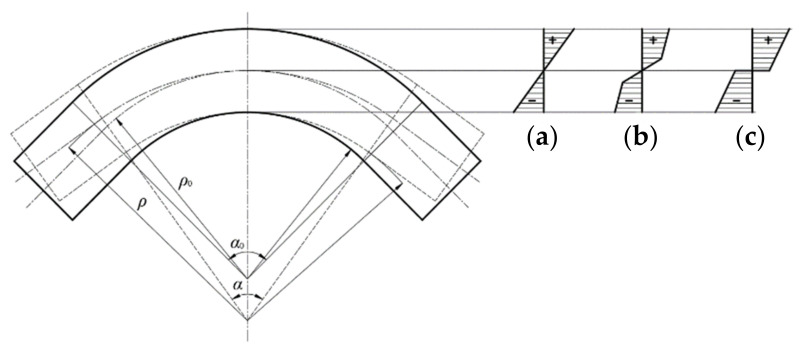
Stress distribution and spring-back during sheet bending: (**a**) elastic bending, (**b**) elastic-plastic bending, (**c**) plastic bending.

The geometric shape and stress distribution in the bending process of the sheet are shown in Figure 1. The initial elastic deformation and elastic–plastic deformation give way to plastic deformation as drawing deformation increases. However, due to the material’s elastoplastic deformation characteristic, spring-back occurs after the removal of external loads in both the elastoplastic- and plastic-deforming stages, causing a large change in the bending fillet radius, angle, and other size characteristics, which affects dimension precision and performance [32].

The variation in the bending angle before and after spring-back can be expressed as:

(1) $$\Delta \alpha \;=\;\alpha_{0}\;-\;\alpha$$

where Δ*α* is the variation in the angle of the specimen; *α* is the bending angle of the sheet after spring-back; and *α_0_* is the bending angle of the sheet before spring-back.

The variation of the bending fillet radius of the specimen before and after spring-back can be expressed as:

(2) $$\Delta \rho \;=\;\rho \;-\;\rho_{0}$$

where Δ*ρ* is the variation in the bending fillet radius of the specimen before and after spring-back; *ρ* is the bending fillet radius of the plate after spring-back; and *ρ_0_* is the bending fillet radius of the sheet before spring-back.

Assuming that the length of the neutral layer is constant, the relationship between the angle and the fillet radius can be expressed as [33]:

(3) $$\alpha_{0}\;=\;\frac{\rho }{\rho_{0}}\;A$$

The relationship between the variation in angle and the variation in the bending fillet radius can be expressed as:

(4) $$\Delta \rho \;=\;\frac{\rho_{0}}{\alpha }\;\Delta \alpha$$

However, the spring-back of the sheet is related not only to the geometric shape but also to the yield strength *σ_s_*, the material hardening exponent *n*, the elastic modulus *E*, and other parameters of material performance. However, the parameters are changed under the CEF. Thus, a systematic study of the effect of the CEF on the parameters is required.

## 3. Experimental Materials and Scheme

### 3.1. Experimental Material

The material used for the tests was TC2 titanium alloy. TC2 titanium alloy is a medium-strength neat-α-type titanium alloy with a microstructure of coexisting α and β phases. Under high temperatures, TC2 titanium alloy usually works at 350 °C for a long time and at 750 °C for a short time. It is mainly used in the manufacture of aircraft wings, stabilizers, flaps, and other stressed components. The chemical composition is shown in Table 1 [34], and the thickness is 1.0 mm. The temperature of thermal forming is usually 550–600 °C [35].

### 3.2. Experimental Principle and Scheme

**Figure 2:**
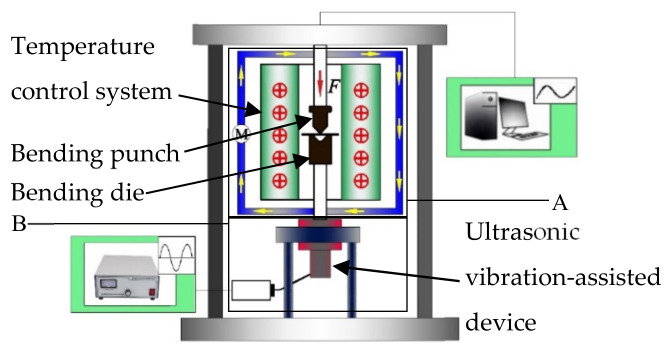
Compound energy field with temperature and ultrasonic vibration-assisted bending experimental device.

As shown in Figure 2, the experimental principle of the forming assisted by the CEF is to control temperature by the temperature control system (A area) and applying ultrasonic vibration by the ultrasonic vibration-assisted device (B area). The compound of temperature and ultrasonic vibration is realized by the superposition and interaction of temperature and ultrasonic vibration.

The tensile tests of the TC2 titanium alloy sheet under different CEF parameters were first carried out to clarify the effect of the CEF on the properties of titanium alloy. Then, the 90° V-shaped mold was selected for the bending experiment under different CEFs, combining the results of the tensile tests to analyze the effect of the CEF on the bending properties of titanium alloy. In the tests, the rate of both tensile tests and bending tests was 0.003 s^−1^. The experimental temperatures were 20 °C, 500 °C, 550 °C, and 600 °C, due to the thermal stability and current investigation of titanium alloy [34]. Ultrasonic vibration with power of 1.0 kW, 1.2 kW, and 1.4 kW, with frequency of 20 kHz, was applied throughout the process of tensile tests and bending tests. In order to validate the consistency of results, each test was carried out six times.

The specimens were first heated to the required temperature and the temperature was held for 10 min to make sure that the tests were carried out in isothermal conditions, in ae furnace controlled by the GW-1200 Electrical Furnace Controller, produced by FangRui Technology Co., Ltd., Changchun, China. Then, the tensile tests and bending tests were carried out.

## 4. Experimental Results and Analysis

### 4.1. Tensile Results and Analysis

**Figure 3:**
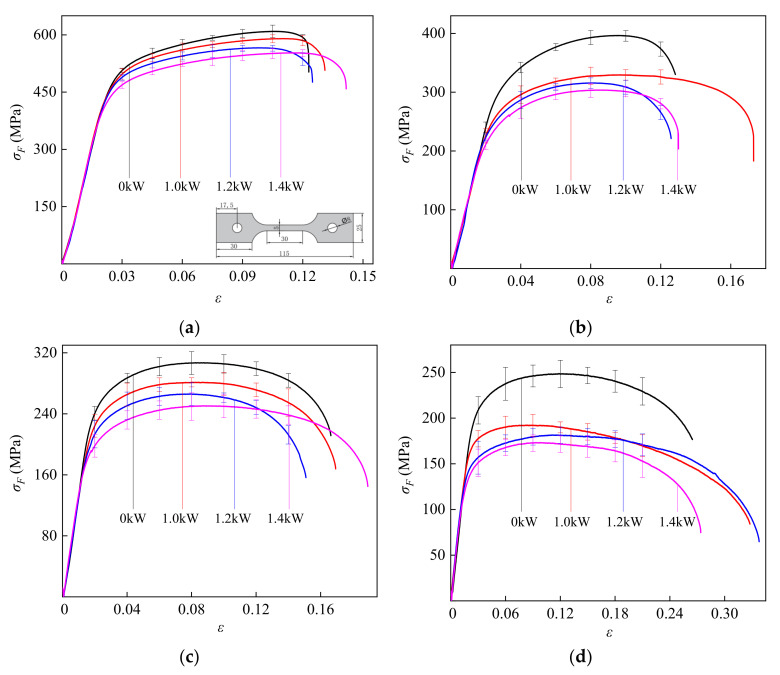
True stress–strain curve of TC2 under the CEF: (**a**) 20 °C; (**b**) 500 °C; (**c**) 550 °C; (**d**) 600 °C.

Figure 3 shows the true stress–strain curve of TC2 under the CEF and the specimen—the size of the tensile specimen is shown in Figure 3a. When there is no ultrasonic vibration (0 kW) as is the case under a single-temperature field, as the temperature increases the strength of TC2 continues to decrease, and the overall stress–strain curve shifts downward. When the temperature is below 550 °C, work-hardening is the main characteristic of the true stress–strain curve. When the temperature reaches 600 °C, the high-temperature softening effect appears. Compared with the single-temperature field, when the deformation enters the plastic deformation stage, the stress–strain curve of TC2 is further reduced under the CEF, indicating that the softening effect has occurred and is maintained during the vibration. In addition, the softening effect increased with an increase in ultrasonic power. Hung et al. performed hot upsetting tests and the results indicated that ultrasonic vibration could considerably reduce the forces during forming [36]. Hung et. al. concluded that the mechanisms of increased temperature and energy absorption of dislocation could affect the material property and cause a reduction in forming forces [37]. The regulations presented in these literatures are in accordance with those observed in our tests.

On the one hand, because ultrasonic vibration helps to increase the capability of surmounting obstacle/dislocation arrays, this leads to a decrease in the dislocation multiplication rate but an increase in the probability of dislocation annihilation [23]. On the other hand, another part of the high-frequency vibration energy will be superimposed with the temperature field, resulting in a reduction in stress. In addition, as shown in Figure 3, the ultimate strain of TC2 fluctuates with the increase in ultrasonic power, caused by the interaction among microstructures [38].

**Figure 4:**
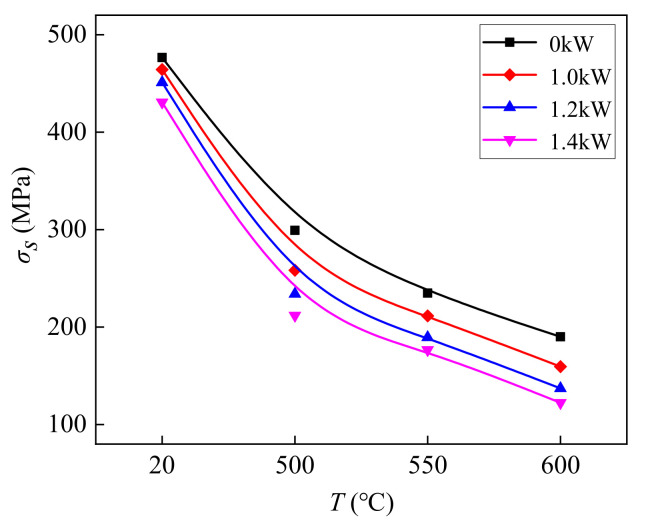
The effect of the CEF on the value of *σ_s_*.

**Figure 5:**
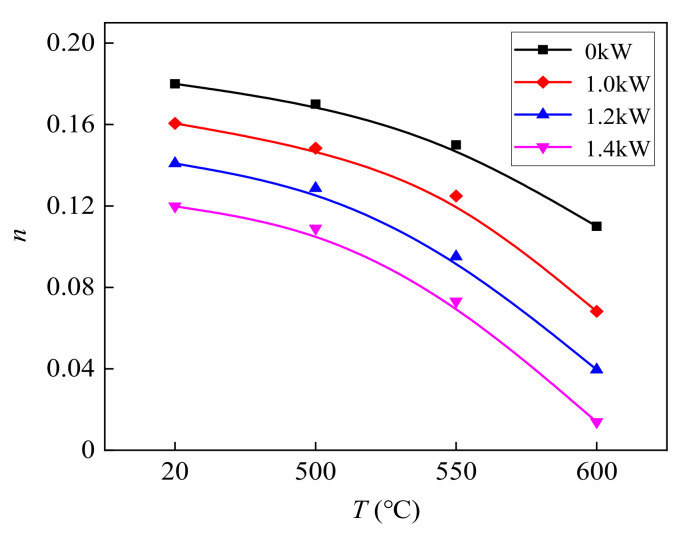
The effect of the CEF on the value of *n*.

**Figure 6:**
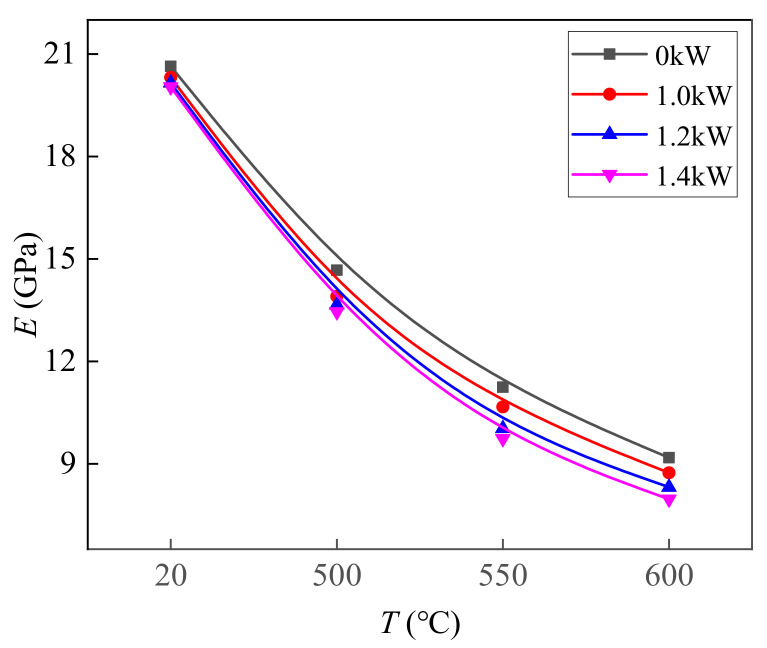
The effect of the CEF on the value of *E*.

Figure 4, Figure 5 and Figure 6 show the values of *σ_s_*, *n*, and *E* of TC2 titanium alloy under different CEFs. The measurements of *σ_s_* were all within the 95% confidence interval of the confidence level. The values of *n* and *E* were calculated with the true stress–strain curve based on the Swift model [39,40]. It can be seen from the figures that the values of *σ_s_*, *E*, and *n* further reduce when the temperature increases. Compared with the single-temperature field, the reduction in the values of *σ_s_* and *n* were larger under the CEF, and the higher the temperature, the greater the reduction in the values of *σ_s_* and *n*. However, the effect of the CEF on the value of *E* was not as obvious as it was on the values of *σ_s_* and *n*.

### 4.2. Bending Results and Analysis

**Figure 7:**
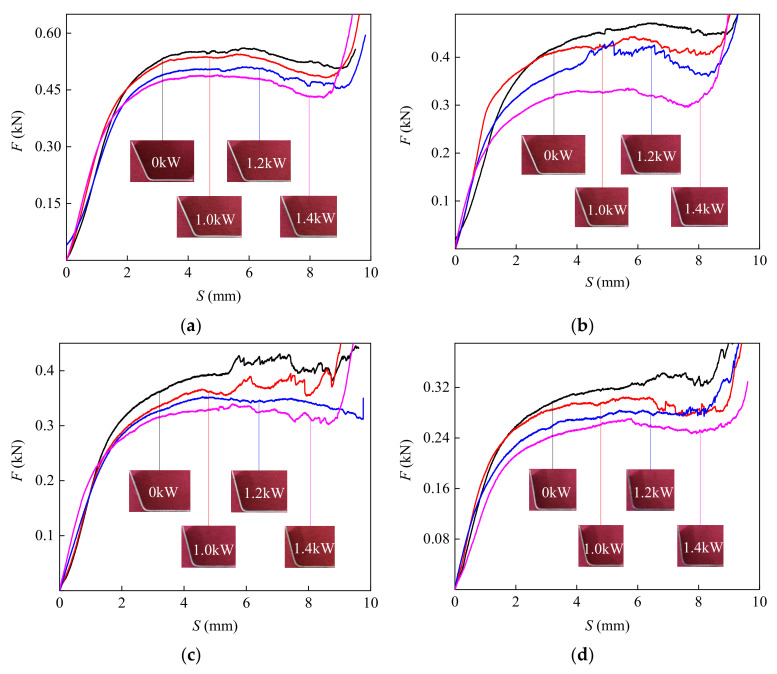
The bending force–punch stroke curve under different CEFs: (**a**) 20 °C; (**b**) 500 °C; (**c**) 550 °C; (**d**) 600 °C.

A 90° V-shaped mold was selected for the bending tests. Figure 7 shows the bending force–punch stroke curve (*F* and, S, respectively) under different CEFs and specimens. The size of the bending specimen was 20 mm × 50 mm. The TC2 titanium alloy is mostly elastically deformed in the early stages of bending deformation, and the bending force grows rapidly with the punch going down. When the punch stroke exceeds 3 mm, the deformation of material changes from elastic deformation to plastic deformation, and the rate of increase in bending force slows down. After the stroke of the punch reaches 9 mm, the bending part fits the mold, which causes the bending force to increase when the deformation continues. At the same time, it can be seen from Figure 6 that the reduction in the bending force–punch stroke under the CEF is larger than it is in the single-temperature field. This is because the ultrasonic energy input increases the high-temperature softening effect. Meanwhile, the instantaneous separation between the specimen and the mold reduces the hindrance to the material flow and the bending force [41].

**Figure 8:**
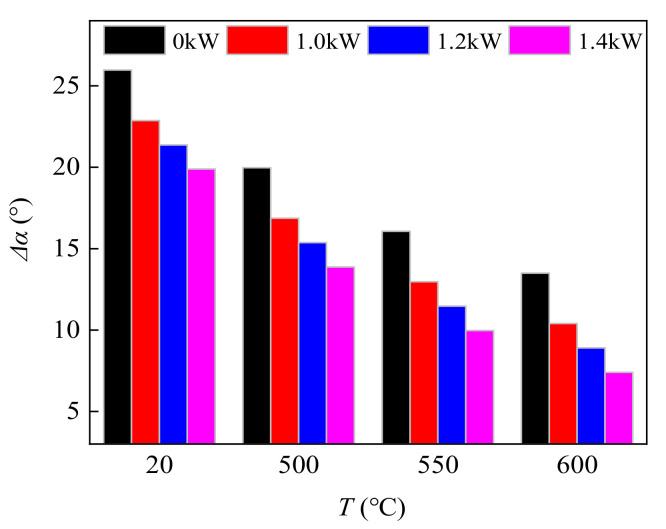
The spring-backs of TC2 bending parts under the CEF.

**Figure 9:**
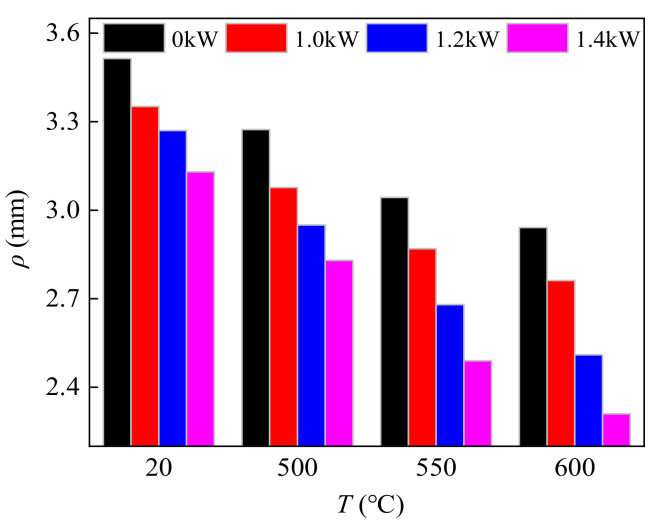
The fillet radius of TC2 bending parts under the CEF.

Figure 8 and Figure 9 show a comparison of spring-back and bending fillet radius under different CEF parameters, which were all within the 95% confidence interval of the confidence level. When the parameters of the CEF were 20 °C/0 kW, 500 °C/0 kW, 550 °C/0 kW, and 600 °C/0 kW (the single-temperature field), the spring-back was 25.97°, 19.97°, 16.07°, and 13.01°, respectively. With the increase in temperature, the spring-back continues to decrease. When ultrasonic power was 1.0 kW, the parameters of the CEF were 20 °C/1.0 kW, 500 °C/1.0 kW, 550 °C/1.0 kW, and 600 °C/1.0 kW, and the spring-back of the bending specimen was 22.87°, 16.87°, 12.97°, and 9.4°, respectively. The reduction was 11.9%, 15.5%, 19.2%, 35.1%, respectively. When ultrasonic power increased to 1.4 kW, the parameters of the CEF were 20 °C/1.4 kW, 500 °C/1.4 kW, 550 °C/1.4 kW, and 600 °C/1.4 kW and the spring-back of the banding specimen was 19.89°, 13.87°, 9.97°, and 7.4°, respectively. Compared with the single-temperature field, the reduction was 23.41%, 30.54%, 37.95%, and 45.18%, respectively. When compared, the effect of inhibiting spring-back of the TC2 titanium alloy under the CEF with 550 °C/1.0 kW was consistent with the effect under the single-temperature field at 600 °C. Moreover, the effect of inhibiting the spring-back of the TC2 titanium alloy under the CEF with 550 °C/1.4 kW was consistent with the effect under the single-temperature field at 600 °C. It can be seen that the effect of the CEF on the bending fillet radius is similar to the spring-back shown in the comparison between Figure 8 and Figure 9. Therefore, under the same temperature conditions, the combination of the temperature field and ultrasonic vibration can further improve the bending properties and the quality of the bending specimen of the TC2 titanium alloy. Alternatively, for the same bending specimen of the TC2 titanium alloy, the combination of the temperature field and ultrasonic vibration can lower the forming temperature, which is helpful in reducing production costs.

### 4.3. Effect of the CEF on Microstructure

To further explore the mechanism of the CEF on the bending properties of the TC2 titanium alloy, the samples were embedded in mosaic powder, then polished to achieve the requirement of no scratches. After corrosion for about 90 s by Kroll’s Reagent, which consisted of 2% HF, 6% HNO_3_, and 92% H_2_O, the samples were observed by the TESCAN MIRA3 scanning microscope (SEM), produced by TESCAN CHINA, Ltd. Then the grain size was manually measured using the intercept method according to ASTM E112 [42]. The average grain size was obtained by measuring three different areas in the sample.

**Figure 10:**
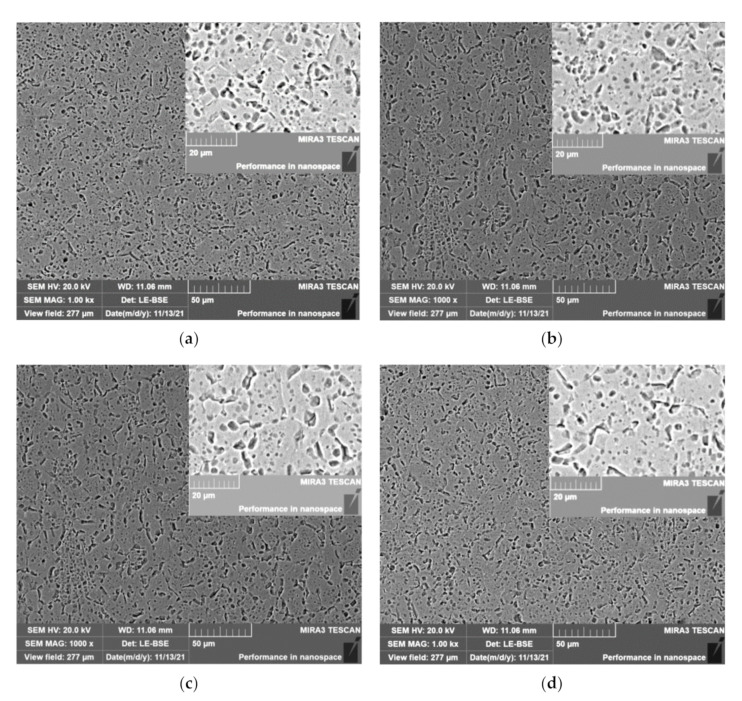
The structure of the TC2 titanium alloy under the CEF: (**a**) 500 °C/0 kW; (**b**) 500 °C/1.0 kW; (**c**) 600 °C/0 kW; (**d**) 600 °C/1.0 kW.

Figure 10 shows the metallographic structure of the TC2 titanium alloy under the CEF. It can be seen that the metallographic structure changes with the change of the parameters of the CEF. Compared with the single-temperature field, the average grain size is larger under the CEF. For example, under the single-temperature field, when the temperatures are 500 °C and 600 °C, the average grain sizes are about 10.4 μm and 10.8 μm, respectively. However, when ultrasonic power is 1.0 kW, the average grain sizes are about 11.5 μm and 12.3 μm under temperatures of 500 °C and 600 °C, respectively. The results further demonstrate that ultrasonic vibration can improve the high-temperature softening effect, in accordance with the results obtained by Wang [23] and Huang [26].

## 5. Conclusions

1. The results of the tensile tests of TC2 under the CEF show that, compared with a single-temperature field, the strength and the material-hardening exponent decrease due to the high-temperature softening effect increased by the CEF. Therefore, the CEF can effectively enhance the plastic formability of the sheet, and the effect increases with the increase in ultrasonic energy.
2. The results of the bending tests for TC2 under the CEF show that, compared with a single-temperature field, the CEF can reduce the bending force and spring-back of the TC2 parts and effectively increase the quality of bending parts.
3. Microstructure observation of the TC2 titanium alloy shows that grain size increases under the effect of ultrasonic vibration, which further increases the high-temperature softening effect.
4. The combination of the temperature field and ultrasonic vibration can further improve the mechanical properties and bending properties of titanium, which is helpful in improving the quality and properties of titanium alloy parts.

## Figures and Tables

**Table 1:** Chemical composition of TC2 titanium alloy (%).

Fe	C	N	H	O	Al	Mn	Ti
≤0.30	≤0.08	≤0.05	≤0.012	≤0.15	≤3.5–5.0	≤0.8–2.0	balance

## Data Availability

Not applicable.
